# T Cell Dynamics in COVID-19, Long COVID and Successful Recovery

**DOI:** 10.3390/ijms26157258

**Published:** 2025-07-27

**Authors:** Zoia R. Korobova, Natalia A. Arsentieva, Anastasia A. Butenko, Igor V. Kudryavtsev, Artem A. Rubinstein, Anastasia S. Turenko, Yulia V. Ostankova, Ekaterina V. Boeva, Anastasia A. Knizhnikova, Anna O. Norka, Vadim V. Rassokhin, Nikolay A. Belyakov, Areg A. Totolian

**Affiliations:** 1 Saint Petersburg Pasteur Institute, Mira St. 14, 197101 St. Petersburg, Russia; 2 Faculty of Medicine, First Pavlov State Medical University of St. Petersburg, L’va Tolstogo St. 6-8, 197022 St. Petersburg, Russia; 3 Institute of Experimental Medicine, Akademika Pavlova, 12, 197022 St. Petersburg, Russia

**Keywords:** COVID-19, T cells, TREC, long COVID, post-COVID, convalescents

## Abstract

Despite targeting mainly the respiratory tract, SARS-CoV-2 disrupts T cell homeostasis in ways that may explain both acute lethality and long-term immunological consequences. In this study, we aimed to evaluate the T-cell-mediated chain of immunity and formation of TCR via TREC assessment in COVID-19 and long COVID (LC). For this study, we collected 231 blood samples taken from patients with acute COVID-19 (*n* = 71), convalescents (*n* = 51), people diagnosed with LC (*n* = 63), and healthy volunteers (*n* = 46). With flow cytometry, we assessed levels of CD4+ and CD8+ minor T cell subpopulations (i.e., naïve, central and effector memory cells (CM and EM), Th1, Th2, Th17, Tfh, Tc1, Tc2, Tc17, Tc17.1, and subpopulations of effector cells (pE1, pE2, effector cells)). Additionally, we measured TREC levels. We found distinct changes in immune cell distribution—whilst distribution of major subpopulations of T cells was similar between cohorts, we noted that COVID-19 was associated with a decrease in naïve Th and CTLs, an increase in Th2/Tc2 lymphocyte polarization, an increase in CM cells, and a decrease in effector memory cells 1,3, and TEMRA cells. LC was associated with naïve CTL increase, polarization towards Th2 population, and a decrease in Tc1, Tc2, Em2, 3, 4 cells. We also noted TREC correlating with naïve cells subpopulations. Our findings suggest ongoing immune dysregulation, possibly driven by persistent antigen exposure or tissue migration of effector cells. The positive correlation between TREC levels and naïve T cells in LC patients points to residual thymic activity. The observed Th2/Th17 bias supports the hypothesis that LC involves autoimmune mechanisms, potentially driven by molecular mimicry or loss of immune tolerance.

## 1. Introduction

As of 2025, the World Health Organization (WHO) reports that COVID-19—triggered by the SARS-CoV-2 virus—has caused substantial morbidity and mortality, exceeding 7 million fatalities [1]. Beyond its mortality, the pandemic has exposed critical limitations in existing research of viral immunopathology, particularly the mechanisms underlying severe disease progression. While in most cases the infection is mild, severe infection often causes critical outcomes, e.g., acute respiratory distress syndrome (ARDS) and multi-organ failure. These complications are primarily driven by cytokine storm and hyperinflammation—a dysregulated immune response marked by excessive cytokine release, e.g., interleukin 6 (IL-6), tumor necrosis factor α (TNFα), and interferon γ (IFNγ), leading to widespread tissue damage [2]. Even after recovery, many patients develop long COVID (LC), experiencing persistent symptoms such as fatigue, cognitive dysfunction, and immune dysregulation [3], suggesting not only somatic changes in the host, but also long-term alterations in immunity.

In terms of epidemiology, long COVID is seen more in patients previously hospitalized (50–70%) than in non-hospitalized patients (10–30%), which suggests a link to disease severity [4,5]. It is yet to be discussed whether the vaccination factor affects incidence rates—in the study by Al-Aly et al., LC is noted in 10–12% of vaccinated cases [6,7]. LC is not an age-dependent disease, but the highest percentage of cases occurred in adults between 36 and 50 years [8]. However, long COVID (LC) is mostly seen in individuals who had mild initial infections—a predictable finding, given that most COVID-19 cases fall into this category.

While the exact causes of LC remain unknown, several potential mechanisms have emerged as likely contributors to its persistent (yet unspecified) symptoms. A leading hypothesis centers on viral persistence, with studies detecting SARS-CoV-2 spike protein circulating months after acute infection, potentially driving chronic inflammation [9,10]. This persistence may facilitate immune dysregulation, characterized by exhaustion of T cells, altered memory subsets, and sustained inflammatory responses, even after mild initial infections [11,12,13].

Another related area of research explores viral reactivation, particularly Epstein–Barr virus (EBV) and other herpesviruses, which may reactivate due to COVID-induced immune dysregulation, exacerbating chronic fatigue and other nonspecific symptoms [14,15]. Additionally, microbiome imbalance—marked by reduced microbial diversity and prolonged gut dysbiosis—has been linked to immune dysfunction and may cause the systemic inflammation in LC [16,17,18].

Autoimmunity is also under investigation, with studies reporting pathogenic autoantibodies targeting GPCRs, ACE2, and other autoantigens, possibly due to molecular mimicry or COVID-triggered loss of immune tolerance [19,20,21]. At the same time, neurological mechanisms and impaired circulation may lead to dysautonomia and cognitive deficits [10,22,23,24,25].

Together, these pathways likely interact, creating a complex web of mechanisms that lead to LC. Future research should clarify their contribution and identify targeted therapeutic strategies.

Studies highlight the role of type 2 diabetes, EBV reactivation, the presence of specific autoantibodies [20], connective tissue disorders [26], neurological disorders, and chronic allergic diseases [27] in LC manifestation. However, in nearly 30% of cases, none of these or any other pre-existing factors are seen [8].

Growing data demonstrate that COVID-19 causes significant, long-lasting shifts in immunological regulation, especially affecting T cell dynamics. Unlike influenza or common cold viruses, SARS-CoV-2 triggers aberrant T cell activation, premature exhaustion, and affects differentiation, including differentiation of regulatory T cells (Tregs) followed by the depletion of effector subsets [28]. Such disruptions in immunity may contribute to both acute disease severity—by impairing viral clearance and enforcing inflammation—and chronic post-viral complications, including LC and increased susceptibility to secondary infections. These results imply that SARS-CoV-2 could permanently modify immune function in convalescent patients, even beyond the pandemic.

Even as a respiratory pathogen, SARS-CoV-2 induces T cell dysregulation in ways that may explain both acute lethality and long-term immunological consequences. The primary objective of this study was to evaluate the T cell-mediated chain of immunity and formation of T cellular receptor (TCR) via T cell receptor excision circles (TREC) assessment in COVID-19 and LC.

## 2. Results

**Figure 1:**
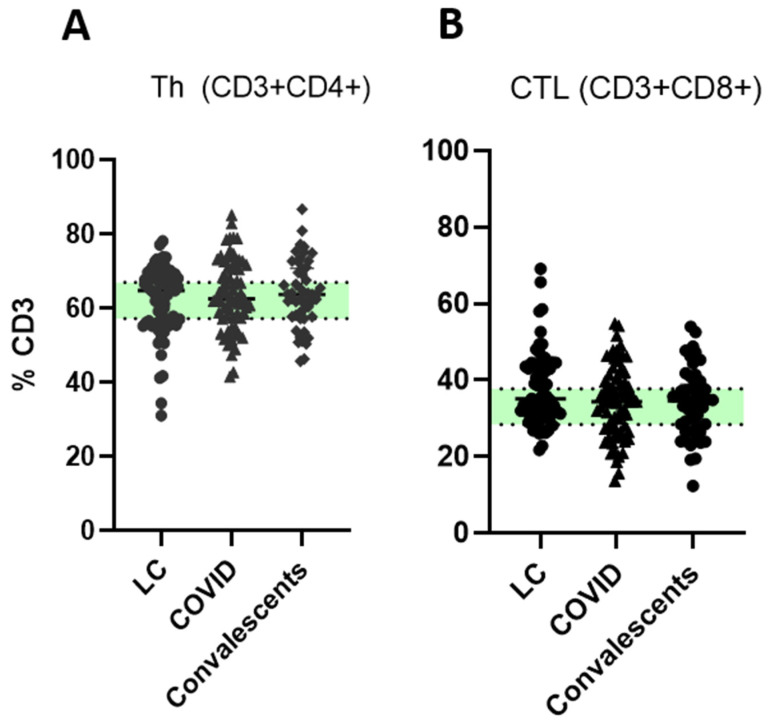
T cells levels in long COVID patients (LC), COVID-19 patients (COVID), and convalescents. (**A**)—Th cells (CD45+CD3+CD4+); (**B**)—cytotoxic T lymphocytes (CTL, CD45+CD3+CD8+); % from CD3+ cells. Green stripe stands for interquartile range (Q25–Q75) for healthy donors. No statistically significant differences between groups were found via Kruskal–Wallis test.

**Figure 2:**
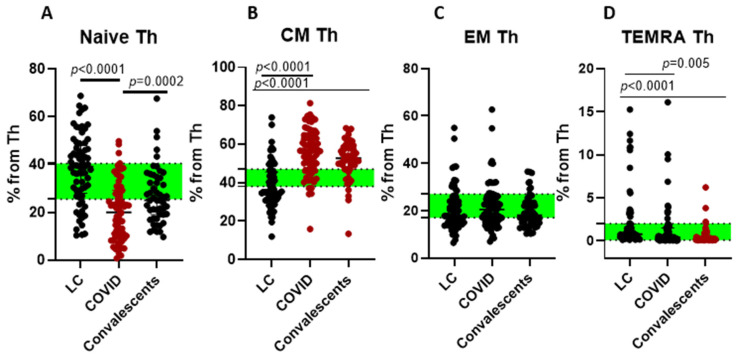
T helper cell differentiation in long COVID patients (LC), COVID-19 patients (COVID), and convalescents. (**A**)—‘naïve’ (CD45RA+CD62L+) T helper cells, (**B**)—central memory (CM/CD45RA−CD62L+), (**C**)—effector memory (EM/CD45RA−CD62L−), and (**D**)—TEMRA (CD45RA+CD62L−) cells (% from CD4+ T helper cells). Green stripe stands for interquartile range (Q25–Q75) for healthy donors. Red color highlights groups demonstrating statistically significant differences when compared to healthy donors based on Kruskal–Wallis test.

While the overall proportions of T helper cells (Th, CD4+) and cytotoxic lymphocytes (CTL, CD8+) were similar across LC, acute COVID-19, and convalescent groups (Figure 1), deeper T cell profiling revealed compositional differences in minor subpopulations. We investigated subsets of CD4+ T helper (Th) cells: ‘naïve’ (CD45RA+CD62L+), central memory (CM/CD45RA−CD62L+), effector memory (EM/CD45RA−CD62L−) and terminally differentiated memory (TEMRA, CD45RA+CD62L−) cells—in patients with acute COVID-19, convalescent individuals, and those with LC, comparing them to healthy donors (HD) (Figure 2).

Our findings revealed substantial alterations in Th cell subsets in acute cases, characterized by a significant reduction in ‘naïve’ Th cells and an increase in CM Th cells compared to HD. Convalescent patients, evaluated 1–3 months post-infection, exhibited a similar Th cell profile to acute cases, with decreased ‘naïve’ Th cells and elevated CM Th cells relative to HD. This pattern may indicate an incomplete recovery of the immune system, as the recent infection could still be influencing lymphocyte homeostasis.

The absence of significant Th cell subset deviations in LC patients versus HD suggests their prolonged symptoms may involve alternative pathological pathways.

**Figure 3:**
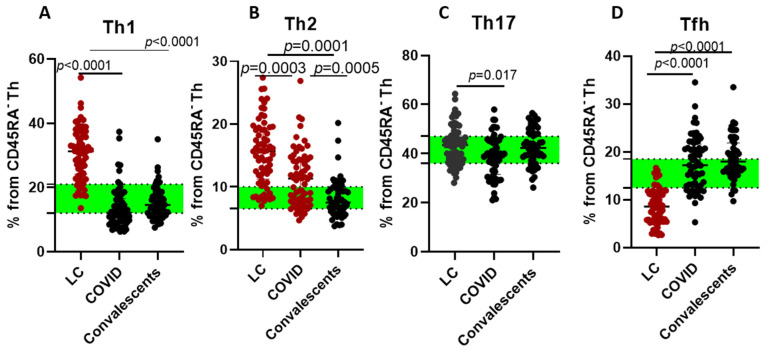
T helper cell polarization in long COVID patients (LC), COVID-19 patients (COVID), and convalescents. (**A**)—Th1 (CXCR5−CXCR3+CCR6−CCR4−), (**B**)—Th2 (CXCR5−CXCR3−CCR6−CCR4+), (**C**)—Th17 (CXCR5−CCR6+), and (**D**)—T follicular helper (Tfh/CXCR5+) cells. Green stripe stands for interquartile range (Q25–Q75) for healthy donors. Red color highlights groups demonstrating statistically significant differences when compared to healthy donors based on Kruskal–Wallis test.

We observed significant shifts in Th cell polarization, particularly in LC patients, who displayed elevated levels of Th1 and Th2 cells compared to HD. Furthermore, long COVID patients displayed reduced levels of Tfh cells, which may indicate impaired humoral immunity or disrupted germinal center responses, potentially contributing to prolonged symptoms (Figure 3).

Notably, Th cell polarization was significantly altered in LC cases, characterized by increased Th1 and Th2 cell frequencies compared to HD. Furthermore, long COVID patients displayed reduced levels of Tfh cells, which may indicate impaired humoral immunity or disrupted germinal center responses, potentially contributing to prolonged symptoms.

**Figure 4:**
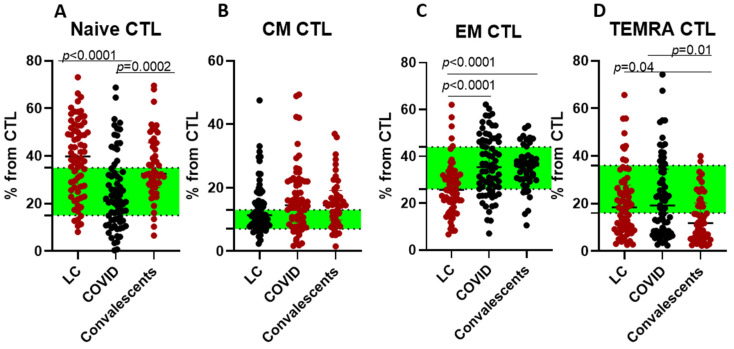
Cytotoxic T lymphocyte (CTL) differentiation in long COVID patients (LC), COVID-19 patients (COVID), and convalescents. (**A**)—‘naïve’ (CD45RA+CD62L+), (**B**)—CM (CD45RA−CD62L+), (**C**)—EM (CD45RA−CD62L−), and (**D**)—TEMRA (CD45RA+CD62L−) cells. Green stripe stands for interquartile range (Q25–Q75) for healthy donors. Red color highlights groups demonstrating statistically significant differences when compared to healthy donors based on Kruskal–Wallis test.

We further investigated cytotoxic lymphocyte (CTL/CD45+CD3+CD8+) subpopulations: ‘naïve’ (CD45RA+CD62L+), CM (CD45RA−CD62L+), EM (CD45RA−CD62L−), and TEMRA (CD45RA+CD62L−) cells (Figure 4).

CM CTLs were significantly elevated in acute infection compared to controls, reflecting T cellular memory activation. However, convalescent and LC patients exhibited a different pattern, with elevated levels of naïve and lowered TEMRA CTLs.

**Figure 5:**
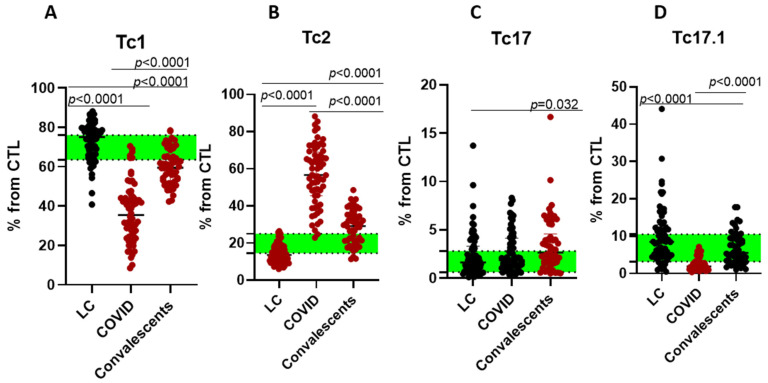
Cytotoxic T lymphocyte (CTL) polarization in long COVID patients (LC), COVID-19 patients (COVID), and convalescents. (**A**)—Tc1 (CXCR3+CCR6−), (**B**)—Tc2 (CXCR3−CCR6−), (**C**)—Tc17 (CXCR3−CCR6+), and (**D**)—Tc17.1 (CXCR3+CCR6+). % from CTL (CD8+ cells). Green stripe stands for interquartile range (Q25–Q75) for healthy donors. Red color highlights groups demonstrating statistically significant differences when compared to healthy donors based on Kruskal–Wallis test.

We also measured levels of CTLs based on their polarization: Tc1 (CXCR3+CCR6−), Tc2 (CXCR3−CCR6−), Tc17 (CXCR3−CCR6+), and Tc17.1 (CXCR3+CCR6+), as shown in Figure 5.

**Figure 6:**
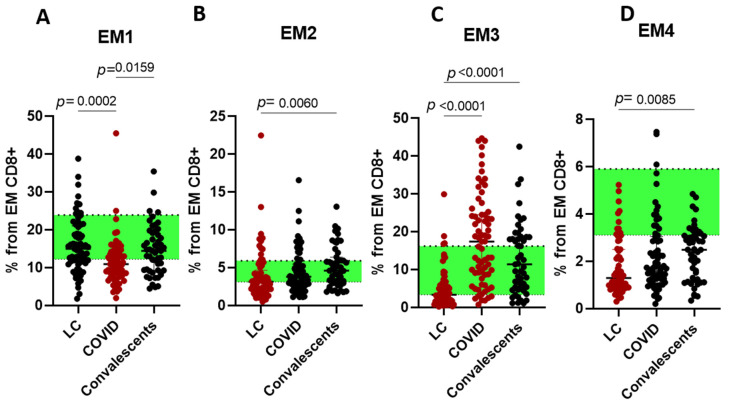
Cytotoxic T effector memory cells subsets in long COVID patients (LC), COVID-19 patients (COVID), and convalescents. (**A**)—EM CD8+ T cell were subdivided into EM1 (CD27+CD28+), (**B**)—EM2 (CD27+CD28−), (**C**)—EM3 (CD27−CD28−), and (**D**)—EM4 (CD27−CD28+) subsets. % from EM CD8+ cells. Green stripe stands for interquartile range (Q25–Q75) for healthy donors. Red color highlights groups demonstrating statistically significant differences when compared to healthy donors based on Kruskal–Wallis test.

Acute-phase patients exhibited reduced Tc1/Tc17.1 but elevated Tc2 frequencies. Convalescents maintained Tc1 suppression with persistent Tc2 increases, whereas LC cases showed significant Tc2 depletion. For the next step, we assessed EM and TEMRA CD8+ T cell subsets with different patterns of CD27 and CD28 expression. EM CD8+ T cells were subdivided into EM1 (CD27+CD28+), EM2 (CD27+CD28−), EM3 (CD27−CD28−), and EM4 (CD27−CD28+) subsets, respectively (Figure 6).

Acute phase was associated with lowered EM1 and increased EM3. Convalescents demonstrated restoration of cells distribution, close to the healthy donors. LC was followed by a decrease in EM2, EM3, and EM4 CD8+ T cell subsets.

**Figure 7:**
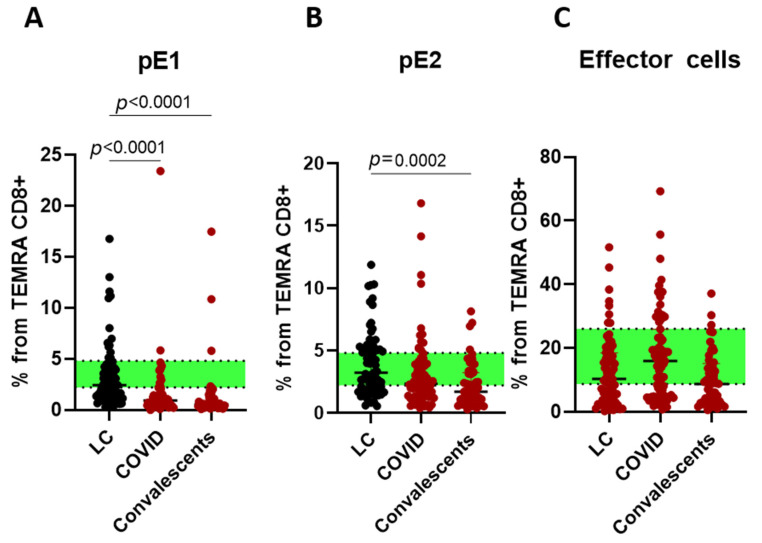
Cytotoxic TEMRA cells subsets in long COVID patients (LC), COVID-19 patients (COVID), and convalescents. (**A**)—pE1 (CD27+CD28+), (**B**)—pE2 (CD27+CD28−), and (**C**)—effector cells (CD27–CD28–). Green stripe stands for interquartile range (Q25–Q75) for healthy donors. Red color highlights groups demonstrating statistically significant differences when compared to healthy donors based on Kruskal–Wallis test.

TEMRA CD8+ T cells were subdivided into CD27+CD28+ pE1 (pre-effector type 1 cells), CD27+CD28− pE2 (pre-effector type 2 cells), and CD27–CD28– E (effector cells) subsets, respectively (Figure 7).

Acute-phase COVID and convalescents demonstrated a decrease in TEMRA populations, while LC was associated with a decrease in effector cells in favor of other populations.

TREC (T-cell receptor excision circle) quantification serves as a biomarker for evaluating thymopoiesis and naive T-cell replenishment, as these episomal DNA fragments are exclusively generated during intrathymic TCR rearrangement and dilute with peripheral T-cell proliferation. Given our prior observations of significantly reduced TREC levels in severe acute COVID-19 patients—indicating thymic suppression during critical illness—we measured TREC concentrations across convalescent, LC, and control cohorts to determine whether impaired thymic output contributes to persistent immune dysregulation.

For reference values in TREC measurements, we used age groups as suggested by the manufacturer [29] (Table 1).

All results for LC patients were within reference values (above lower reference limit) indicating normal formation of T cellular receptor reservoir despite imbalanced T cellular responses.

**Figure 8:**
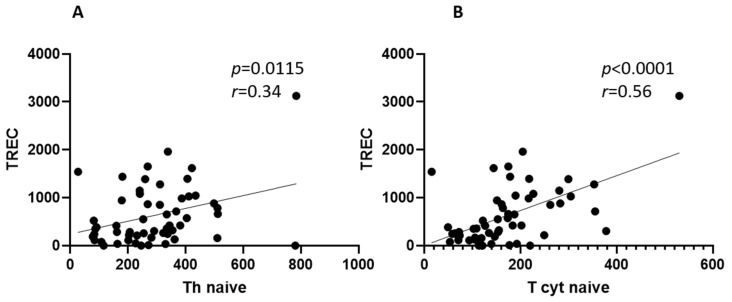
Correlational analysis (Pearson’s r test) of TREC levels (copies × 10^5^) and ‘naïve’ Th cells (**A**) and ‘naïve’ T cytotoxic lymphocytes (**B**) in long COVID patients.

Since we previously reported lower TREC levels in severe acute infection compared to moderate disease [30], we extended this analysis to LC patients, using TREC as a measure of thymic activity and naïve T-cell regeneration (Figure 8).

Our analysis revealed that TREC levels positively correlated with both ‘naïve’ Th (*r* = 0.34, *p* = 0.011) and ‘naïve’ cytotoxic T cell frequencies (*r* = 0.56, *p* < 0.0001) in LC patients and were higher than in the acute stage of the disease and in healthy donors (*p* < 0.05), but were within reference values.

## 3. Discussion

During acute SARS-CoV-2 infection, adaptive T cellular responses play a critical role in viral clearance and disease severity. Research demonstrates an association between disease severity and T cellular lymphopenia, particularly in CD4+ and CD8+ T cells, alongside elevated exhaustion markers (PD-1, TIM-3). Active SARS-CoV-2-specific CD8+ T cell responses with cytotoxic potential are associated with less severe clinical manifestations [31].

Research on immune dysregulation in LC patients with initially mild acute infection has identified persistent T cell abnormalities lasting ≥13 months. These include elevated exhaustion markers (e.g., PD1+ central memory cells), alongside reduced CD4+ and CD8+ effector memory populations, suggesting chronic immune activation and impaired viral clearance mechanisms [13].

T helper lymphocytes (CD4+) are essential for coordinating immune responses via promoting B cell antibody production and CD8+ T cell activation. Protective immunity against SARS-CoV-2 correlates strongly with adequate CD4+ T cell responses, which orchestrate both humoral immunity through antibody generation and long-term immunological memory. SARS-CoV-2-specific CD4+ T cells persist across disease stages, showing Th1 polarization during acute infection and into convalescence, as demonstrated by multiple immunological studies [32].

Circulating Tfh (cTfh) cells correlate with neutralizing antibody production post-vaccination. mRNA vaccines (e.g., Pfizer-BioNTech, Moderna) induce strong Tfh activation, enhancing germinal center reactions and durable immunity. Tfh subsets (CXCR3^+^ vs. CXCR3^−^) may influence antibody quality, with Th1-polarized Tfh supporting IgG responses [33]. Dysregulated Tfh responses are linked to disease severity. Mild cases show functional Tfh-B cell interactions. Severe COVID-19 exhibits aberrant Tfh activation, potentially driving cytokine storms or inadequate antibody responses. Autoantibody production in LC may involve persistent Tfh activity. Some studies report prolonged CXCR5^+^ PD-1^+^ Tfh in LC patients, suggesting chronic germinal center stimulation [34].

Although CD8+ T cells mediate essential antiviral defenses via perforin/granzyme cytotoxic mechanisms, their functional impairment or persistent activation could affect the immune dysregulations observed in long COVID. In acute infection, robust SARS-CoV-2-specific CD8+ T cell responses are associated with milder disease [31]. However, in severe COVID-19, CD8+ T cells often exhibit functional exhaustion, marked by reduced cytotoxicity [35]. The persistence of exhausted or aberrantly activated CD8+ T cell subsets, alongside elevated naive and CM populations, may reflect ongoing immune dysregulation in LC, potentially driving chronic symptoms. Notably, similar T-cell defects have been observed in other post-viral syndromes, suggesting a shared mechanism of immunological dysfunction [36]. Further studies are needed to determine whether these shifts represent failed viral clearance or persistent antigen exposure.

Notably, the expected Th1/Tc1-dominated response—typically associated with antiviral defense—was inhibited. Instead, we observed a trend toward Th2, Tc2, and Th17/Tc17 polarization [37]. We noted similar effects within our previous study [38]. This deviation from canonical antiviral immunity may reflect several mechanisms of COVID-19: for instance, viral invasion due to inhibition of major histocompatibility complex (MHC) I expression [39].

Additionally, we found an increase in ‘naïve’ CD8+ T cells, suggesting altered Tcell differentiation and homeostasis. Prolonged immune dysfunction persisted within 8 months since the infection and was followed by a lack of ‘naïve’ T and B cells and an increase in interferon expression [11]. And as COVID-19 is often followed by cytokine storm, immune disruptions potentially start early as the infection develops [40]. Emerging evidence strongly implicates pre-existing systemic inflammation as a critical predisposing factor for LC. Studies indicate that elevated baseline levels of pro-inflammatory markers, such as IL-6, TNF-α, and CRP, are significantly associated with an increased risk of developing persistent post-acute complications following SARS-CoV-2 infection [41]. This provides a permissive environment where the acute viral insult triggers an exaggerated and dysregulated immune response. Elevated cytokine levels (both peripheral and central) drive the diverse symptomatology of LC, contributing to endothelial dysfunction, microvascular injury, neuroinflammation, and immune cell exhaustion [42].

Persistent T cell abnormalities may contribute to LC development. For instance, bias towards Th2 or Th17 can be a contributing factor towards autoimmune mechanisms associated with COVID-19 severe course and LC. Several new-onset autoantibodies were noted after acute COVID-19, potentially driven by Th2-associated B cell responses reviewed in [43].

SARS-CoV-2-specific T cells remain elevated in some LC patients, suggesting chronic immune activation [44]. Exhausted T-cell phenotypes (e.g., PD-1+, CD57+) are linked to prolonged symptoms [45]. Reduced ‘naïve’ T cells and expanded cytotoxic CD8+ T cells are observed, possibly reflecting persistent antigen exposure often seen in over 12 months since the infection [46]. LC patients exhibited persistent alterations in regulatory T cell proportions, suggesting a potential link to immune-associated sequelae [47]. In younger COVID-19 patients, reduced TREC levels correlated with ARDS and mortality, and were negatively associated with the neutrophil-to-lymphocyte ratio [48].

Patients with COVID-19 demonstrated lowered levels of TREC molecules [49]. In the study by Savchenko, prominent correlations were seen in survivors of the infection between ‘naïve’ T cells and TREC molecules [50], which corresponds to our findings. Another study by Zurochka et al. demonstrated lowered TREC levels in people with 6–12 months history of COVID-19 [51]. The association between TREC numbers and naïve T cells in LC patients suggests residual thymic activity, possibly compensating for peripheral T cell losses during acute infection. However, the higher TREC levels in LC compared to healthy donors raise questions about a delayed or exaggerated rebound in thymopoiesis, as seen in other post-viral states [34]. Whether this reflects adaptive recovery or a dysregulation requires further discussion.

**Figure 9:**
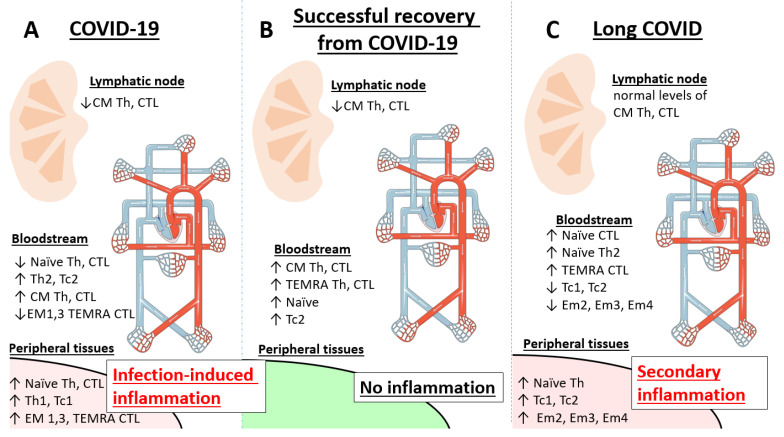
Potential mechanisms of Th/Tc subpopulation dynamics in COVID-19, recovered patients and long COVID. In acute infection (**A**), subpopulations detected in the bloodstream reflect inflammatory processes in tissues, affected by COVID-19. In the convalescence period (**B**), most of the cells in the blood stream are residual from the active inflammatory process. However, in long COVID (**C**), effector cells may potentially return to tissues, where inflammatory sites of non-infectious origin are present.

Based on our findings and existing literature data, we suggest the concept for the underlying autoimmunity in LC (Figure 9). The observed depletion of effector memory and TEMRA cells in peripheral blood may reflect their migration to inflamed tissues, where they could contribute to localized immune dysregulation—even in the absence of overt inflammation. This redistribution suggests compartmentalized immune disruption (peripheral tissues vs. lymphoid organs) rather than systemic depletion. In contrast, central memory T cells remain stable, with levels resembling healthy donors, as CM cells primarily reside in lymphoid organs and are less prone to tissue trafficking.

Co-existing Th1 and Th2 cytokine elevations (IFN-γ/TNF-α and IL-4/IL-13, respectively) may indicate pathogenic bystander activation, mirroring patterns observed in autoimmune disorders where nonspecific inflammatory responses enforce the immune dysregulation. This cytokine imbalance could drive chronic inflammation, contributing to LC symptoms such as fatigue and neural and cognitive dysfunction via systemic cytokine responses. Furthermore, as we previously reported regarding LC immunity, alterations [52] in transitional B cells suggest dysregulated B cell tolerance checkpoints, which may promote autoantibody production (e.g., against GPCRs or interferons) and exacerbate LC pathology.

Together, these mechanisms paint a picture of immune dysregulation leading to T-cell redistribution, aberrant Th1/Th2 activation, and B-cell dysfunction, all of which may highlight the multisystemic nature of LC.

## 4. Materials and Methods

### 4.1. Studied Cohorts

The research was conducted across multiple medical institutions in Saint Petersburg, including the Saint Petersburg Pasteur Institute, Pavlov First Saint Petersburg State Medical University, and the North-Western Scientific and Clinical Center named after L.G. Sokolov. Ethical approval was granted by the Saint Petersburg Pasteur Institute’s Ethical Committee (Protocol #67, 28 April 2021 and Protocol #84, 16 February 2023), and all participants provided written informed consent.

For our study, we collected 231 blood samples taken from patients with acute COVID-19 (*n* = 71), convalescents (*n* = 51), people diagnosed with LC (*n* = 63), and healthy volunteers (*n* = 46). Baseline patient demographic information is presented in Table 2.

Patients with acute COVID-19 were admitted to the specialized COVID-19 Department at the First Saint Petersburg State Pavlov Medical University in 2020–2022. Their infection was caused by the original SARS-CoV-2 genetic strain. The median age of participants was 60 years (Q25–Q75—46–70), with a gender distribution of 25 (35.2%) males and 46 (64.8%) females. Diagnosis was established through clinical assessment and included the following symptoms: fever, fatigue, myalgia, arthralgia, cough, and CT-confirmed pneumonia. Another major criterion for diagnosis was qualitative PCR detection of SARS-CoV-2 RNA, in accordance with the current Russian Ministry of Healthcare’s COVID-19 Guidelines [53], adapted from WHO recommendations [54]. Disease severity was classified as moderate in 49 (69.1%) cases and severe in 22 (31.0%). At discharge, only 3 (4.2%) patients had achieved full recovery, while 35 (49.2%) exhibited only relative clinical and radiological improvement. However, 29 (40.8%) showed no significant resolution of lung tissue damage, and 4 (5.6%) succumbed to complications of the infection. Blood samples were obtained at a median of 7 days (Q25–Q75—4–12) after symptom onset.

The convalescent cohort (*n* = 51) consisted of individuals who had recovered from COVID-19 within 28–35 days before sample collection. Participants had a median age of 32 years (Q25–Q75—26–38), with 21 (41.2%) males and 30 (58.8%) females. At the time of sampling, all patients were asymptomatic, tested negative for SARS-CoV-2 via RT-PCR, and had detectable anti-SARS-CoV-2 IgG antibodies. Based on disease severity during acute infection, 24 (47.1%) had mild, 20 (39.2%) moderate, and 7 (13.7%) severe COVID-19. Blood samples were collected at a median of 73 days (Q25–Q75—62–95) post-symptom onset.

Individuals with LC (*n* = 63), defined by persistent psychoneurological symptoms (such as cognitive impairment assessed via the Montreal Cognitive Assessment, and anxiety/depression measured by the Hospital Anxiety and Depression Scale) alongside somatic complaints (including fatigue and dyspnea lasting over 12 weeks post-COVID-19). Inclusion criteria required a prior PCR-confirmed COVID-19 diagnosis and an age range of 18–60 years. The median age was 38 (29–48), with a predominance of female participants (79%, *n* = 50 vs. 21% male, *n* = 13). In most cases (85%), patients had experienced mild acute COVID-19, while 15% reported moderate or severe illness. Recurrent COVID-19 infections were reported in 82% of participants, and only 41% had received prior COVID-19 vaccination. Participants frequently reported poor quality of life due to hair loss (57.1%), bad appetite (52.4%), nonspecific abdominal pain (46%), and blood pressure fluctuations (100%). Additionally, 74.6% had comorbid non-infectious chronic conditions in remission, such as gastrointestinal disorders (31.7%), cardiovascular diseases (6.3%), and renal/urinary tract issues (9.5%). A subset (15 individuals) had a BMI exceeding 25 kg/m^2^.

The study also included 46 healthy controls (22 males, 24 females), whose peripheral blood samples were obtained prior to the COVID-19 pandemic. The control group had a median age of 42 years (Q25–Q75—35–48).

Given the nonspecific nature of LC and the diversity of the studied cohorts, results of this study should be interpreted with caution.

### 4.2. Sample Collection

Peripheral blood samples (5 mL) were obtained from each patient before any treatment using VACUETTE^®^ K3 EDTA tubes (Griener Bio-One, Kremsmünster, Austria). For TREC assessment, leukocyte fraction of venous peripheral blood was frozen at −80 °C.

### 4.3. T Cell Immunophenotyping by Flow Cytometry

Immunophenotyping was conducted within 6 h of blood collection.

For immunophenotyping of CD4+ and CD8+ T cell subset maturation stages, the following combination of fluorescent-labeled antibodies (all from Beckman Coulter, Brea, CA, USA) were used: anti-CD57 FITC, anti-CD62L ECD, anti-CD28 PC5.5, anti-CD27 PC7, anti-CD4 APC, anti-CD8 APC-AF700, CD3-APC-AF750, anti-CD45RA Pacific Blue, and anti-CD45 Krome Orange.

For immunophenotyping of ‘polarized’ CD4+ and CD8+ T cell subsets, the following combination of antibodies were employed: CD45RA-FITC, CD62L-PE, CD3-APC-AF750, CD4-PacB (Beckman Coulter, Brea, CA, USA), CXCR5-PerCP/Cy5.5, CCR6-PE/Cy7, CXCR3-APC, CCR4-BV510 (BioLegend, San Diego, CA, USA).

Earlier, we described the gating strategy for T cell subset maturation stages and the major ‘polarized’ T cell subsets [55]. The cellular subsets and phenotyping strategy are presented in Table 3.

In total, 200 μL of whole blood was stained with the antibody cocktail (optimized per manufacturer guidelines) in the dark at room temperature for 15 min. Erythrocyte lysis was performed using 2 mL VersaLyse Lysing Solution (Beckman Coulter, Brea, CA, USA) supplemented with 50 μL IOTest 3 Fixative Solution (15 min incubation, dark, RT). Cells were washed with PBS, centrifuged (330× *g*, 7 min), and resuspended in 500 μL PBS containing 2% neutral formalin (Sigma-Aldrich, Burlington, MA, USA). Samples were analyzed on an Acea Novocyte flow cytometer (Agilent Technologies, Santa Clara, CA, USA).

### 4.4. TREC Assessment

After centrifugation, the tube contents yield three distinct layers: an upper plasma layer, a lower erythrocyte/granulocyte pellet, and a buffy coat (leukocyte ring) at the interphase. The leukocyte ring (WBC fraction) was carefully aspirated, washed to remove residual platelets, and processed for DNA extraction. This method ensures high-purity leukocyte isolation, which is critical for accurate TREC quantification in T-cell subpopulations. To assess levels of TREC molecules, DNA was extracted from 250 μL of the WBC fraction using the commercial RIBO prep DNA extraction kit (Central Research Institute of Epidemiology, Moscow, Russia). After that, real-time PCR was performed for all DNA samples with simultaneous amplification of target TREC DNA fragments. Quantitative assessment of the content of TREC molecules was performed using the method of constructing standard curves using the TREC/KREC-AMP PS test system (Saint Petersburg Pasteur Institute, Russia) [56]. Reference TREC levels were determined by the manufacturer [29].

### 4.5. Statistical Analysis

In this study, statistical analysis was conducted using Prism 8.0 (Dotmatics, Boston, MA, USA) and Microsoft Excel 2013 (Microsoft Corporation, Redmond, WA, USA). To determine the appropriate statistical tests, we performed normality and log-normality Kolmogorov–Smirnov test. Non-parametric statistical analyses were employed for group comparisons: the Mann–Whitney U test for pairwise comparisons and the Kruskal–Wallis test for multi-group analyses, with a significance threshold set at *p* < 0.05. Correlational analysis was performed to evaluate relationships between non-parametric variables (Pearson’s r), ensuring a comprehensive approach to our data interpretation, with model strength based on Chaddock scale for correlational analysis interpretation.

## 5. Conclusions

While most clinical studies of COVID-19 immunity focus on major T-cell populations, this study represents the comprehensive immune profiling of COVID-19 patients not only in the acute stage of the disease, but also across mid-convalescence (1–3 months since the infection) and long COVID phases.

Our findings reveal significant alterations in T-cell subsets, particularly in helper (Th) and cytotoxic (CTL) populations, which persist beyond the acute phase of infection and may contribute to the pathogenesis of LC. Acute COVID-19 patients exhibited a certain defect in naïve Th cells and an increase in CM Th cells, a pattern that persisted in convalescents but normalized in LC patients. This suggests that while Th cell homeostasis may eventually stabilize, the prolonged dysregulation during recovery could play a role in persistent symptoms.

LC patients displayed elevated Th1 and Th2 responses alongside reduced Tfh cells, indicating potential disruptions in antibodies formation and germinal center function. These imbalances may contribute to chronic inflammation and autoantibody production, which are hallmarks of LC. Acute infection was marked by an expansion of CM CTLs, while LC patients showed elevated naïve and TEMRA CTLs with reduced effector memory (EM) subsets. This shift suggests either impaired viral clearance or ongoing immune dysregulation, possibly driven by persistent antigen exposure or tissue migration of effector cells. The positive correlation between TREC levels and naïve T-cell frequencies in LC patients points to residual thymic activity, possibly compensating for peripheral losses during acute infection. However, the higher TREC levels compared to healthy donors raise questions about delayed or exaggerated thymic rebound, warranting further investigation. The observed Th2/Th17 bias supports the hypothesis that LC involves autoimmune mechanisms, potentially driven by molecular mimicry or loss of immune tolerance. Our findings underscore the complexity of immune dysregulation in LC, highlighting the potential need for targeted therapeutic strategies.

## Figures and Tables

**Table 1:** Levels of TREC (copies × 10^5^) in long COVID patients (LC) based on age group.

Age Group	Reference (Me, Q25–Q75)	LC Cohort (Me, Q25–Q75)
18–29	553.3, 44.9–2135	874.4, 528.2–1361
30–39	252.7, 23.6–1597.0	612.3, 335–995.5
40–49	191.3, 18.3–1098	177, 18.4–336.5
50–59	131.1, 13.9–1543	187.8, 76.7–246.1
60–69	74.9, 12.5–1715	540.3, 34.5–1046
>70	44.7, 11.4–683.1	n/a

**Table 2:** Baseline characteristics of studied cohorts.

	COVID-19	Convalescents	LC	HD
*n*	71	51	63	46
Age (Me, Q25–Q75)	60, 46–70	32, 26–38	38, 29–48	
Sex distribution (%)	35.2% males, 64.8% females	41.2% males, 58.8% females	21% male, 79% female	47.8% male, 52.2 female
Major symptoms	Fever, fatigue, myalgia, arthralgia, couch, CT-confirmed pneumonia	no	Psychoneurological symptoms (cognitive impairment, anxiety/depression), fatigue, dyspnea	no
SARS-CoV-2 RNA PCR	yes	no	no	no
SARS-CoV-2 IgG	yes/no	yes	yes	no

**Table 3:** Flow cytometry-based phenotypes of T helper and cytotoxic T lymphocyte subsets.

Subpopulation	Phenotype	% from	Subpopulation	Phenotype	% from
T Helper Cells (Th), CD45+CD3+CD4+		Lymphocytes (CD45+)	Cytotoxic T Lymphocytes (CTL) CD45+CD3+CD8+		
Naïve	CD45RA+CD62L+	Th (CD4+)	Naïve	CD45RA+CD62L+	CTL (CD8+)
CM	CD45RA−CD62L+	Th (CD4+)	CM	CD45RA−CD62L+	CTL (CD8+)
EM	CD45RA−CD62L−	Th (CD4+)	EM	CD45RA−CD62L−	CTL (CD8+)
TEMRA	CD45RA+CD62L−	Th (CD4+)	TEMRA	CD45RA+CD62L−	CTL (CD8+)
Th1	CXCR5−CXCR3+CCR6−CCR4−	Th (CD4+)	Tc1	CXCR3+CCR6−	CTL (CD8+)
Th2	CXCR5−CXCR3−CCR6−CCR4+	Th (CD4+)	Tc2	CXCR3−CCR6−	CTL (CD8+)
Th17	CXCR5−CCR6+	Th (CD4+)	Tc17	CXCR3−CCR6+	CTL (CD8+)
T follicular helpers (Tfh)	CXCR5+	Th (CD4+)	Tc17.1	CXCR3+CCR6+	CTL (CD8+)
-	-	-	EM1	CD27+CD28+	EM CTL (CD45RA−CD62L)
-	-	-	EM2	CD27+CD28−	EM CTL (CD45RA−CD62L)
-	-	-	EM3	CD27−CD28−	EM CTL (CD45RA−CD62L)
-	-	-	EM4	CD27−CD28+	EM CTL (CD45RA−CD62L)
-	-	-	Pre Effector cells 1 (pE1)	CD27+CD28−	TEMRA (CD45RA+CD62L−)
-	-	-	Pre Effector cells 2 (pE2)	CD27+CD28−	TEMRA (CD45RA+CD62L−)
			Effector cells	CD27−CD28−	TEMRA (CD45RA+CD62L−)

Notes: EM—effector memory; CM—central memory; TEMRA—terminally differentiated memory cells.

## Data Availability

The data presented in this study are available on request from the corresponding author due to institutional privacy policy.

## Abbreviations

The following abbreviations are used in this manuscript:

CM	Central memory
EM	Effector memory
KREC	Kappa-deleting recombination excision circles
LC	Long COVID
MHC	Major histocompatibility complex
Tc	Cytotoxic T cells
TCR	T cellular receptor
TEMRA	Terminally differentiated effector memory T cells
Th	Helper T cells
TREC	T cell receptor excision circles
